# The Modulation of Adaptive Immune Responses by Bacterial Zwitterionic Polysaccharides

**DOI:** 10.1155/2010/917075

**Published:** 2010-12-22

**Authors:** Tom Li Stephen, Laura Groneck, Wiltrud Maria Kalka-Moll

**Affiliations:** ^1^Department of Medicine, University of Pennsylvania, Philadelphia, PA 19104, USA; ^2^Institute for Medical Microbiology, Immunology, and Hygiene, Medical Centre, University of Cologne, Goldenfelsstraße 19-21, 50935 Cologne, Germany; ^3^Department of Internal Medicine, Evangelisches Krankenhaus, Weyertal, 50931 Cologne, Germany; ^4^MVZ Dr. Stein + Kollegen, Wallstraße 10, 41061 Moenchengladbach, Germany

## Abstract

The detection of pathogen-derived molecules as foreign particles by adaptive immune cells triggers T and B lymphocytes to mount protective cellular and humoral responses, respectively. Recent immunological advances elucidated that proteins and some lipids are the principle biological molecules that induce protective T cell responses during microbial infections. Polysaccharides are important components of microbial pathogens and many vaccines. However, research concerning the activation of the adaptive immune system by polysaccharides gained interest only recently. Traditionally, polysaccharides were considered to be T cell-independent antigens that did not directly activate T cells or induce protective immune responses. Here, we review several recent advances in “carbohydrate immunobiology”. A group of bacterial polysaccharides that are known as “zwitterionic polysaccharides (ZPSs)” were recently identified as potent immune modulators. The immunomodulatory effect of ZPSs required antigen processing and presentation by antigen presenting cells, the activation of CD4 T cells and subpopulations of CD8 T cells and the modulation of host cytokine responses. In this review, we also discuss the potential use of these unique immunomodulatory ZPSs in new vaccination strategies against chronic inflammatory conditions, autoimmunity, infectious diseases, allergies and asthmatic conditions.

## 1. Introduction

The primary role of the immune system is to protect the host from microbial invasions and infections. While nonspecific, innate immune mechanisms mediate the first stage of transient protection against the invading pathogens, more advanced adaptive immune mechanisms prevent microbial invasions and infections by activating antigen-specific T and B cells. Adaptive immune responses are superior to innate immunity because they provide pathogen specificity and immunological memory, which effectively prevents future reinfections of the host by the same pathogens [1]. 

The generation of effective adaptive immune responses to infectious agents requires the recognition of invading microbial pathogens by T and B cells. While B cells can recognize the native protein antigens on pathogen surfaces, T cells do not directly recognize the surface molecules of pathogens. T cells specifically recognize the processed antigenic components of invading pathogens [2]. An exception to this are the superantigens, a group of powerful antigens occurring in various bacteria and viruses that bind outside of the normal T cell receptor (TCR) site, reacting with multiple TCR molecules and activating T cells nonspecifically [3, 4]. Antigen presenting cells (APCs) are a group of specialized cells that efficiently process and present both self-and nonself pathogen-derived antigens to T cells for recognition [5–7]. Professional APCs, such as dendritic cells (DCs), macrophages, and B cells, efficiently process pathogen-derived molecules in their cellular compartments to generate antigenic fragments that can be loaded into major histocompatibility molecules (MHCs). MHCs then present the antigenic fragments to T cells for recognition [5]. While CD4+ T cells recognize antigens presented by MHC class II (MHCII) molecules, antigens presented by MHC class I (MHCI) are recognized by CD8+ T cells. Even though microbial pathogens are composed of proteins, carbohydrates, lipids, and nucleic acids, only protein antigens are currently believed to be processed and presented on MHC molecules by APCs for T-cell recognition and activation [5, 8–10]. Several studies that used TCR transgenic mouse models show that T cells are positively selected by peptide-MHC complexes in the thymic cortex, and the availability of the crystal structures for TCR-peptide-MHC-complexes [11] reinforces the theory that T cells only recognize peptide antigens. The results of these studies further confirmed the notion that only proteinaceous antigens induce adaptive T cell responses. However, important questions remain as to whether nonproteinaceous antigens can activate T cells in an MHC-restricted manner. The discovery of the CD1-mediated presentation of glycolipids to *γδ*T cells [12, 13] and *αβ* TCR+ NK T cells [14] suggests that T cells can also recognize non-protein molecules. However, carbohydrate antigens, which are important components of pathogenic organisms and conjugated vaccines, were considered to be T cell-independent antigens that are not presented on MHC molecules and do not induce T cell activation [15]. However, recent advances in research of antigen processing and presentation and carbohydrate immunobiology are challenging this traditional concept.

## 2. Bacterial Zwitterionic Polysaccharides Activate CD4 T Cells

**Figure 1:**
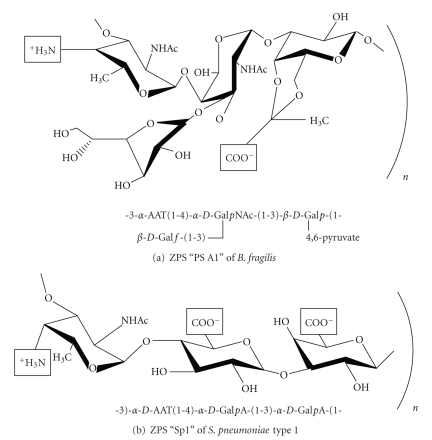
Structure of ZPSs. (a) Repeating sugar molecules of *Bacteroides fragilis* PS A1 carrying zwitterionic charge motives. (b) ZPS Sp1 of *S. pneumoniae* serotype 1.

Most of the naturally occurring polysaccharide molecules are composed of negatively charged (anionic) sugar molecules. These anionic sugar molecules fail to activate T cells and do not induce B-cell antibody isotype switching [16]. However, Dennis Kasper's laboratory identified a group of bacterial polysaccharides that carry both positive and negative charges in the same repeating sugar molecules and are thus called “zwitterionic polysaccharides (ZPSs)” [17–22]. Pathogenic strains of bacteria, such as *Bacteroides fragilis *(*B. fragilis*), *Streptococcus pneumoniae *(*S. pneumoniae*), and *Staphylococcus aureus* (S*. aureus*), produce ZPSs [22–24]. The capsular polysaccharide antigens PSA of *B. fragilis* (NCTC 9343 and 638R) [22] and Sp1 of *S. pneumoniae* serotype 1 [24] are the most widely studied ZPSs. The biological activities of ZPSs from different bacterial strains are very similar, and therefore, in this article, we use the term ZPS to represent all of them. ZPSs display an extended right-handed helix structure in which two repeating sugar units per turn form grooves with positive charges exposed on the outer surface (Figure 1) [24, 25]. Due to this unique structure, ZPSs possess immunomodulatory activities, unlike other polysaccharides [26–28]. Experimental studies in rats and mice have shown that intraperitoneal challenges with ZPSs and the sterile cecal content (SCC) adjuvant induced pathogenic conditions, such as intra-abdominal abscesses [26, 29]. SCC alone failed to induce abscess formation. However, subcutaneous vaccinations using only ZPSs prior to the intraperitoneal challenges prevented abscess inductions [30]. Intra-abdominal abscess formation, which commonly occurs during secondary peritonitis and abdominal surgeries, is a protective mechanism used by the body to limit the spread of microbial pathogens. In the initial and subsequent studies of ZPSs, ZPS-induced pathologies were shown to be T-cell dependent. In those experiments, T-cell-deficient mice were unable to form abscesses after inoculations with ZPSs [31, 32]. Further experiments with *α*/*β* TCR-knockout mice revealed that abscess formations were dependent on *α*/*β* TCR+ T cells [33]. The activation of CD4 T cells is required for ZPS-mediated intra-abdominal abscess induction because mice lacking CD4 T cells failed to develop abscesses [33]. As abscess formation was inhibited by the transfer of T cells from animals that were immunized with ZPS [34], these results suggest that abscess inductions and protection in the presence of ZPS are likely mediated by T-cell components of the adaptive immune system. 

The immunomodulatory effects of ZPSs require both positive and negative charge motifs as neutralization of a single charge motif abrogates the biological activities of ZPSs [26]. The abscess-inducing capabilities of *B. fragilis*, which carries a mutant non-ZPS molecule, are severely attenuated [35]. On the other hand, the conversion of a non-zwitterionic molecule into a zwitterion makes the molecule biologically active [27, 36]. These studies suggest that unlike most other polysaccharides, ZPSs represent a unique group of carbohydrates that are able to activate components of the adaptive immune system.

## 3. The Processing and Presentation of ZPSs for CD4 T-Cell Activation

The activation of T cells by proteinaceous antigens requires antigen processing and presentation on MHC molecules followed by TCR recognition of peptide-MHC complexes on the surfaces of APCs [5, 6]. Alternatively, mitogenic bacterial superantigens can nonspecifically activate T cells by crosslinking the MHCs of APCs and the V*β* subunits of TCRs on cell surfaces independently of the intracellular antigen processing and presentation pathway. The intracellular antigen-processing pathways can be divided into exogenous and endogenous pathways [6], though recent discoveries of antigen crosspresentation and autophagy mechanisms show significant crosstalk between these two pathways [9]. Because the antigen crosspresentation and autophagy mechanisms have been reviewed elsewhere [9] and the importance of these pathways in ZPS processing and presentation is currently unknown, this article mainly focuses on the conventional exogenous pathway. In the exogenous pathway, extracellular antigens that are taken up into the cell by pinocytosis or receptor-mediated endocytosis are targeted to endosomes, which then mature and fuse with lysosomes. In the lysosomes, protein antigens are degraded by lysosomal acid hydrolase enzymes into small antigenic peptides that can be loaded onto MHCII molecules with the help of MHCII-like molecules (DM in mice and HLA-DM in humans) (reviewed in [5]). The peptide-MHCII complexes are then transported through tubule-vesicular structures to the cell surface, where they are positioned for TCR recognition by CD4 T cells [37, 38]. In the endogenous pathway, intracellular foreign antigens and self-proteins are processed by proteasomes. The generated peptide fragments are then transported to the endoplasmic reticulum (ER) by the transporter associated with antigen processing (TAP) proteins. In the ER, the peptide fragments are mounted to MHCI molecules [7, 9]. The peptide-MHCI complexes are then transported to the cell surface and recognized by CD8 T cells.

 Recent studies have revealed that ZPSs are processed by several components of the exogenous pathway that are involved in the processing of protein antigens and are discussed below. The recent observation that ZPS-mediated activation of CD4 T cells requires direct contact with MHCII-expressing APCs [24] suggests that an exogenouse antigen processing pathway may exist for ZPSs. Furthermore, the ZPS-mediated activation of CD4 T cells requires the internalization of the polysaccharides into APCs by pinocytosis and endocytosis [39, 40]. Related studies have revealed that the ZPS PSA1 from *B. fragilis* was degraded into small polysaccharide units by nitric oxide (NO) in the endosomal compartments [40]. The ZPS-containing pinosomes or endosomes underwent further maturation and fused with lysosomes [39, 40]. Failure to detect ZPS localization with endoplasmic reticulum markers (TLS and WKM, unpublished data), suggests that these polysaccharides are unlikely processed and presented by the endogenous antigen processing and presentation pathway [40]. In the lysosomes, ZPSs colocalize with MHCII molecules and various protein antigens including ovalbumin [39, 40]. Similar to protein antigens, the acidification of lysosomal compartments, which activates lysosomal acid hydrolases that break down complex protein antigens into smaller antigenic peptides, is required for the ZPS-induced activation of CD4 T cells [24, 40–42]. Interestingly, biochemical analysis revealed that ZPSs bind to MHCII molecules [24, 40, 43], and that this binding requires the zwitterionic charge motifs [40]. The ZPS binding to MHCII molecules requires the catalytic activity of DM, a chaperone molecule that facilitates peptide editing and loading onto MHCII molecules in lysosomes. In the absence of DM, ZPS failed to bind to MHCII molecules [39, 40, 43]. Live cell imaging analysis demonstrated that the ZPS-MHCII complex is transported by a retrograde mechanism through MHCII tubular-vesicular structures from the lysosomes to the cell surfaces of APCs [39]. As expected, the retrograde MHCII-mediated transport of ZPSs from lysosomes to the cell surface was inhibited in the absence of DM molecules [39]. Thus, in the absence of DM chaperone activity, ZPS is neither bound to MHCII nor transported to the cell surface for presentation to CD4 T cells. Taken together, the above studies show that binding of ZPS to MHCII in the lysosomes is not accidental, but is orchestrated by highly specific events.

**Figure 2:**
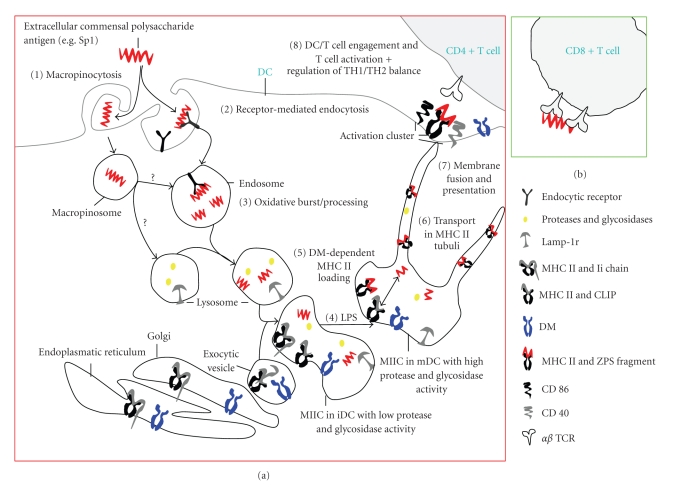
(a) Schematic model illustrating the key steps in the presentation pathway of ZPS antigens in DCs. (1) ZPS antigens are internalized by macropinocytosis through PI3-kinase-dependent mechanism and (2) receptor-mediated endocytosis. (3) In early endosomes, DCs potentially undergo an oxidative burst, including the production of nitric oxide (NO) to process the antigen to lower molecular weight (MW) polysaccharides. ZPS-containing early endosomes fuse with late endosomes and then with lysosomes. Macropinosomes fuse with endosomes or lysosomes. (4) The MHCII protein with Ii chain and the DM molecule are assembled in the endoplasmatic reticulum (ER), transported through the Golgi apparatus, and then budded into exocytic vesicles. ZPS-rich endo/lysosomes fuse with exocytic vesicles, creating a MIIC vesicle carrying MHCII, DM, LAMP-1, proteases, and glycosidases. (5) LPS triggers maturation of iDCs with an increased Ii cleavage to CLIP, DM, and MHCII-antigen-binding activity, and protease and glycosidase activity, which possibly process ZPS to fragments of different lower molecular sizes. DM catalyses antigen exchange of CLIP and other self-peptides with ZPS. ZPS is loaded onto MHCII. (6) ZPS/MHCII complexes are shuttled in tubules originating from lysosomes to the cell surface for (7) fusion with the cell membrane and presentation on the cell surface. (8) Presentation of MHCII/ZPS and costimulatory CD40 and CD86 signals induce DC/T cell engagement and immune responses of CD4 T cells *in vitro* and *in vivo* through the *αβ* TCR. (b) ZPS activation of CD8 T cells by just crosslinking the surface TCRs without MHCI-mediated presentation.

 Contrary to the conventional antigens, bacterial superantigens do not require intracellular processing steps to bind with MHCII [44]. Thus, the requirement for intracellular processing and the MHCII-mediated retrograde transport of ZPSs for APC presentation suggest that ZPSs do not display a superantigen-like activity. In agreement with this observation, ZPS-activated CD4 T cells used a broad V beta (V*β*) TCR repertoire, which is unlike superantigens that use a limited V*β* TCR repertoire [45, 46]. Collectively, these studies revealed a novel MHCII-mediated presentation pathway of ZPSs to CD4 T cells (Figure 2).

While the ZPS processing and presentation and activation of adaptive immune system has recently been studied extensively, relatively little information is available concerning the innate components of the immune system in ZPS-induced immune responses. A recent study by Wang et al. shows that ZPS activates TLR-2, an innate pathogen recognition molecule. ZPS interactions with TLR-2 influence both the induction of iNOS and the upregulation of MHCII and costimulatory molecules associated with ZPS processing and presentation to T cells. In the absence of TLR-2, ZPS-induced secretion of interferon gamma (IFN*γ*) by CD4 T cells and intra-abdominal abscess inductions were significantly reduced [47]. We observed that NF-*κ*B translocation to the nucleus by ZPS Sp1 is inhibited in TLR-2 and TLR-4 knockout cells (unpublished data). Lewis et al. recently reported that ZPS acidification of endosomal compartments enhances the processing of both the polysaccharide and protein antigens and increases the TLR-9 recognition of microbial nucleic acids [42]. Thus, the ZPS-mediated activation of the adaptive immune system is in turn enhanced by the proper ligation of innate pathogen recognition molecules, such as those of the TLR pathway. Since the focus of this article is ZPS modulation of the adaptive immune system, the remaining sections will focus on the adaptive immune responses. 

The presentation of ZPSs by MHCII molecules raises many interesting questions. First, do any lysosomal glycosidase enzymes exist in mammalian cells that can break down ZPSs into small fragments, which can then bind to MHCII? Second, where do ZPSs bind to MHCII molecules? Recent structural and functional studies have shown that unlike MHCI molecules, the open-ended antigen-binding groove of MHCII molecules may accommodate larger antigenic peptides [48–50]. Thus, does DM facilitate the loading of ZPSs exactly into the antigen-binding groove of MHCII? Third, what size ZPS molecules are bound to MHCII molecules? While biochemical studies indicate that ZPSs of approximately 15–20 kDa are associated with MHCII molecules [24, 40], circular dichroism (CD) spectra analysis suggest that a minimum of three repeating sugar molecules are required for the ZPS helical structure and MHCII-binding ability [51]. Thus, the sizes of ZPSs that bind to MHCII are variable, and even the smallest ZPS fragment that is bound to MHCII is much larger than the antigenic peptides that are normally processed and bound to the peptide binding groove of MHCII. This fact raises the possibility that tails of the sugar molecules are outside of the MHCII antigen binding groove and may be hanging on the sides of the MHCII molecule. This conformation can potentially affect the canonical TCR and CD4-coreceptor interactions with the MHCII-antigen complexes. Finally, do any ZPS-specific receptors exist on the surface of APCs? Answering these questions is important for understanding ZPS processing and presentation on MHCII molecules and also for the generation of more-effective polysaccharide-based vaccines to prevent diseases.

## 4. Mechanisms of CD4 T-Cell Activation by ZPS

As described above, due to their unique tertiary structures [51], ZPSs possess immunostimulatory functions that have previously been exclusively attributed to proteinaceous antigens. *In vitro*, the stimulation of human and murine CD4 T cells with ZPSs induces cellular proliferation and inhibits apoptosis [24, 41, 45, 52]. Costimulatory signals are also necessary to raise sufficient T-cell responses to ZPSs because T-cell proliferation was inhibited when CTL4A IgG/B7 CD28 and CD40/CD40L contacts were inhibited [41] (Figure 2). Thus, the activation of CD4 T cells by ZPS is dependent on costimulatory factors. The presentation of fragmented ZPSs by MHCII molecules implies that T-cell activation is mediated by TCR recognition. However, it was unclear how ZPS potentially bound to the TCR. V*β* chain repertoire analyses of ZPS-stimulated T-cell populations showed that a broad repertoire of subfamilies, including all of the V*β* subfamilies, was used rather than specific V*β*  genes [45, 46]. Therefore, the oligoclonal activation of T cells by ZPS seemed probable. Clonotype mapping of T cells that were stimulated *in vitro* with ZPSs showed oligoclonal T-cell expansion [45]. This nonrestricted V*β* usage indicates possible ZPS recognition by the CDR3 antigen-binding domain of the TCR. However, structural analysis, such as X-ray crystallography, is required to validate this hypothesis.

The precise mechanisms of proximal and distal TCR signaling events associated with ZPS recognition are poorly understood. We previously showed that ZPS stimulation upregulates the expression of CD69, an early T-cell activation marker, on CD4 T cells [39]. After TCR stimulation, CD69 is rapidly upregulated by the Ras-MAP kinase signaling pathway [53, 54], suggesting that this pathway may be involved in ZPS-mediated T-cell activation. However, more studies are required to determine if ZPS recognition results in the activation of key proximal TCR signaling molecules, such as Lck, Zap70, Lat, and SLP76 [55]. Moreover, analysis of ZPS-specific T-cell clones is required to increase our understanding of the mechanisms of T-cell activation. Stingele et al. fused *in vitro* activated rat T cells and mouse thymoma cell line BW5147.G.1.4 to generate ZPS-specific T-cell clones [46]. These T-cell clones showed enhanced responses to ZPS stimulation and prevented the intra-abdominal abscess formation in experimental models. However, significant crossreactivity between ZPS subtypes, but not with non-ZPS molecules, was observed among these T-cell clones. The attempts to generate mouse ZPS-specific T-cell clones *in vitro* have not met with success. The difficulties in generating specific T-cell clones could be due to the complex immunomodulatory activities of ZPS, such as the induction of T-cell anergy and the generation of IL-10-producing T cells (discussed below), which suppress the IL-2 production required for expansion of T-cell clones [46, 56]. As an alternative approach, clonotype mapping of abscess-inducing CD4 T cells from mouse models shows the existence of ZPS-specific clones [45]. It is therefore important to know that ablation of ZPS-induced immune suppression, such as IL-10 production and anergy, can expand the above identified ZPS-specific CD4 T-cell clones *ex vivo*.

## 5. ZPS Induces Regulatory CD8 T Cells Upon TCR Crosslinking

**Figure 3:**
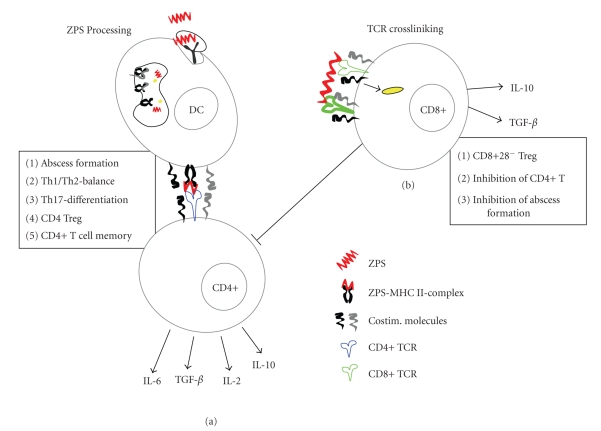
ZPSs modulate CD4 and CD8 T-cell responses. (a) Professional antigen presenting cells such as DCs internalize, process, and present ZPSs on MHCII molecules. The ZPS-MHCII complex is then recognized by TCRs of CD4 T cells in the presence of costimulatory molecules. This results in the activation and differentiation of CD4 T cells. ZPS-activated CD4 T cells produce different cytokines and have diverse immune function such as (1) inducing intraabdominal abscess, (2) helping to balance Th1/Th2 immune responses, (3) inducing the differentiation of proinflammatory IL-17 T cells, (4) inducing IL-10 producing anti-inflammatory CD4 T regs, and (5) differentiate into memory phenotype. (b) In contrast to the CD4 T-cell activation which requires recognition of ZPS-MHCII complex by TCR, ZPS activation of CD8 T cells do not require antigen processing and presentation. ZPS crosslinking of the TCRs activate CD8 T cells. ZPS-activated CD8 T cells differentiate into CD8+CD28^−^ Treg and have different immune modulatory effects such as inhibition of abscess induction and CD4 T-cell activation.

Because most ZPS studies have been primarily focused on CD4 T-cell interactions, the effects of ZPSs on the stimulation of CD8 T cells have only very recently been studied. We showed that ZPSs promote immunosuppressive CD8 T-cell responses [57]. While the absence of CD8 T cells did not inhibit abscess induction [33], their depletion led to the development of significantly larger abscesses [57]. Furthermore, CD8 T cells were localized in the abscess walls. CD8 T cells that invade sites of inflammation have CD28^−^ phenotypes (Figure 3(b)). These CD8+CD28^−^ cells are also CD44lowCTLA4+CD39+ and synthesize IL-10 and TGF*β* upon stimulation with ZPSs, which classifies the cells as immunosuppressive CD8 Treg (CD8 Treg) cells. CD8+CD28^−^ T-cell populations expand in mice that are exposed to ZPSs. ZPS-induced CD8+CD28^−^ immunosuppressive T cells were shown to inhibit non-specific CD4 T-cell responses *in vitro* and ZPS-specific T-cell responses *in vivo*. The adoptive transfer of CD8+ Treg prior to intraperitoneal challenge with ZPS prevented abscess formation [57]. Besides the induction of IL-10-producing CD8+ Treg, ZPS also induces immunosuppressive CD4+ Treg, which produce IL-10 and prevent pathological conditions such as inflammatory bowel disease (IBD) and experimental autoimmune encephalomyelitis (EAE) [58–60]. Thus, the immunomodulatory effects of ZPSs are mainly mediated by the generation of IL-10, an anti-inflammatory cytokine. Besides parasites, viruses and mycobacteria [61], ZPS is the first microbial antigen identified thus far that can induce these CD8+ Treg populations.

 The induction of CD8 Treg by ZPS occurs independently of direct T-cell/APC contact, but CD8 T-cell activation is dependent upon TCR signaling. Stimulation of the cells with ZPSs induced the activation of proximal TCR signaling molecules, such as ZAP70, in CD8 Tregs. In contrast to the proposed oligoclonal CD4 T-cell activation that occurs through the antigen-specific TCR, CD8 T-cell activation is achieved by enhancing TCR crosslinking. Thus, ZPS-mediated activation of CD4 and CD8 TCRs occur through significantly different pathways. In the case of CD4 T cells, ZPS processing and presentation on MHCII is a prerequisite for activation. On the other hand, a subpopulation of CD8 T cells is sufficiently activated by simply crosslinking the TCRs. It will be interesting to explore why the TCRs of CD4 and CD8 T cells display strikingly different ZPS recognition mechanisms. A recent report by Tsai et al. shows that TLR-2 agonists induce the differentiation of CD8 Tregs [62]. Therefore, it is interesting to know that if induction of CD8 Tregs by ZPS also require TLR-2 activation on CD8 T cells. Also, as the CD8+ Treg are generated by TCR crosslinking, it is important to know if different molecular sizes of ZPS influence their expansion. Furthermore, future studies are needed to determine why ZPSs specifically activated CD28^−^ CD8 T-cell populations when the ZPS stimulation of CD4 T cells was CD28-B7-dependent [41].

## 6. ZPS Modulates Cytokines of the Immune System

Intra-abdominal abscess formation is the most important complication that is associated with abdominal surgeries. The ZPS-producing *B. fragilis* bacterium is widely documented in the formation of intra-abdominal abscesses [63, 64]. At the same time, ZPSs have been shown to cause unusual immunomodulatory effects in T-cell-mediated immune protection, which prevented the induction of abscesses. Besides the activation of APCs and CD4 T cells, ZPS-mediated immunomodulation also regulates the production of cytokines and cell adhesion molecules (Figure 3(a)). ZPSs induced the production of TNF*α* and IL-1a on peritoneal cells, which was shown to be important in the induction of abscesses [65, 66]. TNF*α* and IL-1a are required for the transendothelial migration of neutrophils. Inhibiting the production of TNF*α* resulted in the reduction of intercellular adhesion molecule 1 (ICAM-1) on polymorphonuclear cells, which are key players in the formation of intra-abdominal abscesses. The reduction in ICAM-1 inhibited the migration of polymorphonuclear cells to the peritoneal cavity and prevented the development of abscesses [66]. Besides causing the secretion of chemoattractants, such as IL-1, TNF*α* and IL-8 [65], which mediate cellular aggregation to the sites of inflammation, ZPS also induces IL-6, a pleiotropic inflammatory cytokine [52]. IL-6 is a cytokine with both proinflammatory and anti-inflammatory functions and plays important role in T cell stimulation, proliferation and survival by preventing apoptosis [67–70]. In the absence of IL-6, the abscess formation is inhibited, which suggests that the recruitment, activation, and survival of CD4 T cells are required for the induction of abscess formations. We and others have recently demonstrated that ZPS induces the differentiation of Th17 T cells, which secrete the proinflammatory cytokine IL-17 [33, 45]. The differentiation of CD4 T cells into the Th17 lineages requires TGF-*β* and IL-6 (reviewed in [71]). Blocking the production of IL-17 inhibited the induction of abscess formations by ZPSs, suggesting that IL-17 plays critical role in the pathogenesis of ZPS-mediated intra-abdominal abscess formations. While IL-6 stimulation in the presence of TGF-*β* promotes the Th17 T cell development [71], IL-6 *trans*-stimulation of T cells abrogates the development of Foxp-3+ Treg from a naive CD4+CD25^−^ population [72]. Therefore, it is important to know whether besides inducing a proinflammatory immune response, IL-6 also suppresses Treg differentiation during intra-abdominal challenge with ZPS. 

ZPS possesses an unusual biological property that allows it to prevent the induction of intra-abdominal abscesses when it is used in subcutaneous vaccinations. Initial studies have shown that protection from abscess formation was mediated by IL-2 because abscesses were prevalent in mice treated with anti-IL-2 antibodies and ZPS. Furthermore, the adoptive transfer of CD4 T cells from mice that were vaccinated with ZPS prevented the induction of intra-abdominal abscesses in the recipients in an IL-2-dependent manner [73]. Thus, IL-2 is required for the differentiation of ZPS-specific CD4 T cells to effector cells. IL-10 is another cytokine that is implicated in the protection of ZPS-mediated abscess induction [56, 57]. IL-10 is a very potent anti-inflammatory cytokine that limits the inflammatory responses in experimentally infected models [74–76]. In a rodent model for fibrosis, a subpopulation of CD4 T cells were shown to produce the IL-10 upon ZPS immunization [56]. Another study showed that IL-10-producing CD4 T cells, which are CD45RBlow, prevented intestinal inflammation caused by *Helicobacter hepaticus *[58]. We recently showed that upon ZPS immunization, CD8+ Treg populations expanded, produced IL-10, and suppressed the CD4-mediated induction of intra-abdominal abscesses [57]. Besides having specific effects on the formation of ZPS-induced abscesses, the IL-10-producing CD4 Treg populations seemed to broadly protect the mucosal immune system from diseases mediated by microbial pathogens, such as *H. hepaticus*-induced experimental colitis [58, 59]. Therefore, ZPS immunizations could be attractive tools to prevent infections and T cell hyperactivation. Based on this theory, it will be interesting to determine if subcutaneous ZPS vaccinations prevent differentiation of the Th1 and Th17 proinflammatory CD4 T cell populations.

While the unique immunomodulatory effects of ZPS to induce as well as protect the formation of intra-abdominal abscesses has now been known for decades, the underlying cellular and molecular mechanisms are not yet fully understood. As discussed above, intra-abdominal ZPS challenge induces the differentiation of proinflammatory CD4 T cells resulting in abscess formation. However, the same immunogen, when subcutaneously administered without an adjuvant, induces the activation of immunosuppressive CD4 and CD8 Tregs, both secrete immunosuppressive IL-10 molecules and prevent intra-abdominal abscess formation. However, it is interesting to note that both the CD4 and CD8 T cells are recruited to the abscess wall upon intra-abdominal ZPS administration [45, 57], but the induction of immunosuppressive CD4 and CD8 phenotypes were inhibited in the peritoneum during the abscess development. This raises important questions pertaining to the possibility of different APC subsets involved in ZPS presentation in the peritoneal cavity and lymph nodes to induce differential activation of T cell subsets. Upon encounter with exogenously administered ZPS together with SCC adjuvant, ZPS-specific T cell clones that may exist in the immune system (not yet characterized) expand and mediate the intra-abdominal abscess formation. On the other hand, upon subcutaneous administration without adjuvant, ZPS activates the APCs differentially than during intra-abdominal challenge, or activate a different subset of APCs than those involved in ZPS presentation in the peritoneal cavity, resulting in the activation of regulatory T cells. We and others previously reported that ZPS can induce the upregulation of CD86 on APCs [28, 41] *in vitro* and signaling through CD86 is reported in promoting the generation of IL-10-producing T cells [77, 78]. Thus, it will be interesting to know if the differences that occur in the immune responses when ZPS is administered by intraperitoneal or subcutaneous routes are due to differences in the presentation of ZPS antigens by the APCs at these sites. Also, it would be worthwhile to study if subcutaneous administration of ZPS together with a proinflammatory adjuvant can elicit a protective, anti-inflammatory immune response, or ameliorate abscess induction upon subsequent intra-abdominal challenge.

## 7. ZPS Balances the Host Immune System

While the subcutaneous vaccination and intra-abdominal abscess induction studies identified interesting immunomodulatory aspects of ZPSs, it is important to note that ZPSs are continuously presented to the immune system. ZPSs are components of the commensal bacterial flora, such as *B. fragilis* in the gut, *S. aureus* in the skin and mucosa and *S. pneumoniae* in the upper respiratory tract. Accordingly, ZPSs where shown to influence the Th1/Th2 balance of the host immune system. Colonization with *B. fragilis* that contains ZPSs induces a shift of the immune system towards Th1 responses in germ-free mice, which have immune systems that are polarized towards Th2 [28]. *B. fragilis* that produce the mutated form of ZPS (*B. fragilis* ∆PSA), which lacks the zwitterionic charges, failed to induce the Th1 shift of the immune system in germ-free mice. This modulation of the host immune system by ZPS is mediated by IL-12, which is produced by DCs and activates signal transducer and activator of transcription 4 (STAT4) signaling molecules in CD4 T cells to produce Th1 cytokines, such as IFN*γ*  [*28*]. Furthermore, ZPS administration generates CD4 memory T cells with proinflammatory Th1 and Th17 phenotypes [45]. Thus, ZPS presentation by APCs promotes the differentiation of proinflammatory CD4 T cells in a Th2-biased adaptive immune system.

## 8. Clinical Implications

The first murine model that was used for ZPS studies assessed the impact of ZPSs on the formation of intra-abdominal abscesses. Abdominal abscess formation is a major complication associated with abdominal surgeries, endoscopic therapies and inflammatory pathologies that involve mucosal barrier deficiencies, such as inflammatory bowel disease (ulcerative colitis and Crohn's disease) or diverticulitis. *B. fragilis* is one of the most frequently isolated bacteria from abdominal abscesses [64, 79]. Besides the abscesses development, another complication that is frequently associated with abdominal surgeries is adhesion formation. Subcutaneous immunizations with ZPS have been shown to prevent or reduce the formations of abscesses and adhesions in experimental rodent models [26, 56]. These studies also revealed the role of IL-10-producing regulatory CD4 T cells and soluble IL-10 and IL-2 in preventing the formation of intra-abdominal abscesses. Therefore, ZPS injections during abdominal surgeries are very attractive approaches to potentially prevent complications and reduce morbidities, mortalities, and high costs.

In EAE, the murine model for human multiple sclerosis (MS), pathogenesis is mediated by self-reactive T cells that target the myelin basic proteins of the central nervous system [80]. IL-10 has been reported to be a regulatory cytokine in EAE. Elevated levels of IL-10 were associated with disease remission in EAE [81] and MS [82]. Moreover, while IL-10 deficient mice developed severe EAE [83, 84], transgenic mice that overexpressed human IL-10 were resistant to the disease [85]. Importantly, a recent report by Ochoa-Repáraz et al. shows that ZPS can protect the mice from EAE through production of IL-10 producing CD4 Tregs [60]. Thus, ZPS immunizations, which induce IL-10 production by subpopulations of CD4+ and regulatory CD8+ T cells, may have the therapeutic potential to influence the progression of MS. 

Crohn's disease is believed to be caused by T cell immunity abnormalities (e.g., pathologic T cell balances in the mucosal-associated immune system) [86]. ZPSs have been shown to correct the T cell imbalance in germ-free mice [28], and recent studies demonstrated that immunizations with ZPSs from *B. fragilis* prevented inflammatory bowel diseases (IBD) in mice [58, 59]. These ZPS studies enhance our understanding of the underlying immunologic conditions and the interactions between the microbial flora and the host immune system that promote IBD. 

Another widely discussed clinical implication of ZPS usage is in the prevention of allergies ([28] and reviewed in [87]). Briefly, in industrialized nations, improved sanitation conditions and the excessive use of antibiotics and vaccines eliminated many of the commensal and pathogenic flora that were present in our bodies. These communities of microorganisms play critical roles in shaping our immune systems. As a consequence of their elimination, certain populations of people within developed nations are more susceptible to immune system disorders, such as allergies and asthma, which are characterized by Th2 immune responses. Because ZPSs were shown to ameliorate the defective Th2-biased immune systems in germ-free mice, ZPS immunizations might also prevent allergies and asthma. 

A recent study reported that vaccines with synthetic ZPS motifs provided protection against group B streptococcus infections [36]. This implies that ZPSs are vaccine candidates not only against bacterial strains carrying the specific polysaccharide but also to related capsulated bacterial species. Sp1-producing *S. pneumoniae* is still the most frequent cause of bacterial pneumonia, which is often a lethal disease. So far, more than 90 serotypes of S. *pneumonia* have been identified. Two vaccines currently in use to prevent pneumococcal infections are a protein-polysaccharide pneumococcal conjugate vaccine (Prevenar13), and a 23-valent pneumococcal polysaccharide vaccine (PPSV, also known as Pneumovax23) (refer the Center for Disease Control web site, http://www.cdc.gov/ncidod/aip/research/spn.html). Pneumococcal polysaccharide vaccines are highly effective in adults, though not in children, suggesting that without conjugated proteins, carbohydrates alone can induce a protective immune response in adults. Interestingly, both the pneumococcal vaccines also contain the capsular polysaccharide from *S. pneumoniae* serotype 1, the strain which produce the ZPS Sp1 (Prevenar13 web page, http://www.prevnar.com/What-Is-Prevnar13, and Pneumovax23 web page, http://www.merck.com/product/usa/pi_circulars/p/pneumovax_23/pneumovax_pi.pdf).

As the ZPS Sp1 is able to activate CD4 T cells of adaptive immune system, future studies can optimize ZPS concentrations in vaccines to prevent pneumococcal and related bacterial infections. 

Thus, the unusual immunomodulatory properties of ZPSs make them attractive candidates for the treatment of many disease conditions. However, ZPS-based vaccination strategies are still in early experimental stages. To optimize ZPSs as immunoregulatory/vaccine candidates, more research aimed at elucidating the mechanisms of immune modulation by ZPSs is needed.

## 9. Summary

Unlike negatively charged polysaccharides, bacterial polysaccharides with both positively and negatively charged sugar molecules are able to activate CD4 and CD8 T cells of the adaptive immune system. This phenomenon demonstrates that molecules other than proteinaceous antigens are capable of activation of conventional *αβ*T cells. However, the thymic selection mechanisms underlying the *in vivo* generation of this ZPS-specific T cell clones are currently unknown. Development of specific TCR transgenic mouse models are required to understand the selection and development of ZPS-specific T cells. These cells may be more enriched in the mucosae as ZPS-producing bacteria such as *B. fragilis* and *S. pneumoniae* are contained within the commensal flora of the gut and upper respiratory track, respectively. Thus, these ZPS-specific T cells are in constant contact with ZPS presented by APCs of the mucosal immune system. Accordingly, the steady-state presentation of this unique immunomodulatory microbial antigen is beneficial to our immune system as it can play pivotal role in shaping a functionally competent, immune system. However, when ZPSs are accidently introduced into a sterile area, such as the peritoneal cavity during intra-abdominal surgical procedures when abdominal contents can spill into the peritoneal cavity, or by intraperitoneal inoculation with ZPS and a sterile cecal adjutant, a rapid recruitment of ZPS-specific, proinflammatory CD4 T cells may be induced. This recruitment of CD4 T cells together with other innate cells results in the generation of intra-abdominal abscess, a defensive mechanism of the body to contain the infection. Recent studies have shown that ZPS-mediated intra-abdominal abscess induction depends on ZPS processing and presentation on MHCII molecules by APCs for recognition and activation of CD4 T cells. However, future studies are needed to determine how ZPS molecules are bound to MHCII, a paradigm described so far only for protein antigens. Difficulties in crystallizing sugar molecules hamper the study of the structural aspects of ZPS-MHCII interactions as well as those of the TCR-ZPS-MHCII-complex. However, newer approaches such as molecular docking [88] may be useful in resolving the technical difficulties in analyzing ZPS-MHCII interactions. 

The unique immunomodulatory property of ZPS is its ability to prevent the intra-abdominal abscess development when the experimental models are subcutaneously immunized with ZPS prior to intraperitoneal inoculation. During subcutaneous inoculation, ZPSs induce anti-inflammatory, immunosuppressive T cells. This population includes both the regulatory CD4 and CD8 T cells, which secrete the immunosuppressive cytokine IL-10. Thus, the same immunogen can elicit both proinflammatory and anti-inflammatory responses dependent upon the route and mode of administration. While the induction of proinflammatory responses and abscess induction requires ZPS inoculation with sterile cecal content as an adjuvant, induction of immunosuppressive responses by ZPS does not require an adjuvant. Thus, besides the possible differences in antigen presentation by APCs in eliciting these opposing biological responses, the role mediated by adjuvant also needs to be studied in more detail. Moreover, further studies are required to understand the mechanisms of APC-independent activation of CD8 T regs upon subcutaneous ZPS immunization.

Regardless of the unanswered questions, the accumulating data on ZPS is changing our understanding about carbohydrate immunology. We now know that endosome acidification by ZPS can enhance APC functions, such as the processing and presentation of antigens. This is in disagreement with the traditional view that carbohydrates accumulate in the lysosomes and inhibit antigen processing and presentation. ZPS studies have also shown that besides proteins, carbohydrates can also bind to MHCII, an observation that opens the door for further studies in the area of antigen processing and presentation. Moreover, this paves the way for the design of new carbohydrate vaccines against infectious organisms. Additionally, ZPS studies have revealed that some carbohydrates antigens can activate both the CD4 and CD8 T cells of the adaptive immune system and induce proinflammatory or immunosuppressive responses upon administration through different routes. The ability to induce the production of anti-inflammatory cytokines, such as IL-10, makes ZPSs an attractive molecule for the generation of vaccines that can potentially prevent bacterial infections, inflammatory bowel diseases, and autoimmune diseases, such as multiple sclerosis.
